# Classification of Systemic Lupus Erythematosus Using Raman Spectroscopy of Blood and Automated Computational Detection Methods: A Novel Tool for Future Diagnostic Testing

**DOI:** 10.3390/diagnostics12123158

**Published:** 2022-12-14

**Authors:** Emma L. Callery, Camilo L. M. Morais, Lucy Nugent, Anthony W. Rowbottom

**Affiliations:** 1 Department of Immunology, Royal Preston Hospital, Preston PR2 9HT, UK; 2 Institute of Chemistry, Federal University of Rio Grande do Norte, Natal 59072-970, Brazil; 3 Department of Immunology, Whiston Hospital, Prescot L35 5DR, UK; 4 School of Medicine, University of Central Lancashire, Preston PR1 2HE, UK

**Keywords:** SLE, Raman spectroscopy, immunology, rheumatology, diagnostic, multivariate analysis, biospectroscopy, dsDNA, biomarker, molecular, clinical

## Abstract

The aim of this study was to explore the proof of concept for using Raman spectroscopy as a diagnostic platform in the setting of systemic lupus erythematosus (SLE). We sought to identify unique Raman signatures in serum blood samples to successfully segregate SLE patients from healthy controls (HC). In addition, a retrospective audit was undertaken to assess the clinical utility of current testing platforms used to detect anti-double stranded DNA (dsDNA) antibodies (*n* = 600). We examined 234 Raman spectra to investigate key variances between SLE patients (*n* = 8) and HC (*n* = 4). Multi-variant analysis and classification model construction was achieved using principal component analysis (PCA), PCA-linear discriminant analysis and partial least squares-discriminant analysis (PLS-DA). We achieved the successful segregation of Raman spectra from SLE patients and healthy controls (*p*-value < 0.0001). Classification models built using PLS-DA demonstrated outstanding performance characteristics with 99% accuracy, 100% sensitivity and 99% specificity. Twelve statistically significant (*p*-value < 0.001) wavenumbers were identified as potential diagnostic spectral markers. Molecular assignments related to proteins and DNA demonstrated significant Raman intensity changes between SLE and HC groups. These wavenumbers may serve as future biomarkers and offer further insight into the pathogenesis of SLE. Our audit confirmed previously reported inconsistencies between two key methodologies used to detect anti-dsDNA, highlighting the need for improved laboratory testing for SLE. Raman spectroscopy has demonstrated powerful performance characteristics in this proof-of-concept study, setting the foundations for future translation into the clinical setting.

## 1. Introduction

Autoantibodies associated with systemic lupus erythematosus (SLE) are clinically important and have been used historically in the diagnosis and monitoring of disease [1]. The detection of antinuclear antibodies (ANA) and anti-double stranded DNA (dsDNA) antibodies have been included within the laboratory parameters for the classification of SLE in the American College of Rheumatology (ACR) and the Systemic Lupus International Collaborating Clinics (SLICC) criteria [1,2]. However, there are several points of contention relating to the inclusion of these criterions in the classification of SLE. Importantly, ANA are not considered disease-causing and are also present in up to 20% of normal healthy individuals. A wide spectrum of molecular specificities encompass the umbrella term ANA; homogenous ANA patterns directed against dsDNA and histone have the highest sensitivity for SLE (93–95%) [3]. Despite its high clinical sensitivity, the presence of a dsDNA antibody is not a unifying marker in the serological assessment of SLE. A critical review of anti-dsDNA antibodies as a classification marker for SLE can be found by Rekvig [4].

### 1.1. Current Laboratory Testing for SLE-Associated Autoantibodies

The average diagnostic delay for SLE in the UK is 6.4 years [5]. This is likely due to the highly variable clinical and laboratory presentations of the disease. Moreover, within the SLE patient population, current testing methods demonstrate inconsistencies between results and clinical diagnosis, both between patients and between routine testing platforms available for detection of anti-dsDNA antibodies.

A consideration of the differences between assays should be made when evaluating the value of immunological testing in SLE [6]. The two main methods commonly used in routine clinical laboratories to detect anti-dsDNA antibodies are quantitative enzyme-linked immunoassays (ELIA) and semi-quantitative Crithidia Luciliae indirect immunofluorescence (CLIFT). Farr radioimmunoassay (RIA), a third method which has historically been considered the ‘gold standard’ for dsDNA testing, is being phased out of clinical laboratories and replaced with the above-mentioned non-radioactive assays [7].

Existing assays to detect anti-dsDNA antibodies differ in sensitivity, specificity and antigenic substrate. Despite the introduction of a WHO International Standard for anti-dsDNA (Wo/80) and subsequent WHO Reference Reagent (15/174), it has not been possible to establish commutability between the available assays. This is likely due to variabilities in the detection of low-affinity antibodies across assay platforms. The inconsistency between assays increases the potential for the misinterpretation of laboratory results, delayed diagnosis, and a profound negative impact on patient QOL. In light of this, current guidance recommends confirmatory testing for dsDNA with a high specificity assay [8]. In the present study, we have confirmed these findings through a retrospective audit examining six hundred determinations for anti-dsDNA antibodies. We demonstrate that although current testing pathways (ELIA ± CLIFT) have high clinical importance, the inconsistencies identified reinforce the need to develop a novel diagnostic platform for SLE.

### 1.2. Novel Testing Platforms for Clinical Laboratories

New discoveries and advances in technologies mean that we can explore new laboratory approaches for the investigation of complex disorders. However, the identification of novel diagnostic biomarkers with sufficient diagnostic and prognostic accuracy for SLE has not surpassed the performance of current methods [9]. In healthcare, there is an ongoing drive to develop a low cost and simple-to-process analytical platform that can be routinely accessible for patient testing both in the laboratory and at the bedside. One potential and innovative candidate that could enable this is vibrational spectroscopy.

Vibrational spectroscopy is an umbrella term to describe the techniques used to produce a unique spectral read-out, or molecular ‘fingerprint’ of a sample following excitation with light. The unique molecular fingerprint of a sample relates to its biomolecular constituents (i.e., proteins, lipids, nucleic acids, carbohydrates) and is generated from the vibrations of the chemical bonds in these molecules. The molecular fingerprint of a sample will change due to the presence of disease; therefore, vibrational spectroscopy is a well-placed candidate for the study of pathological processes and development of a novel diagnostic platform [10,11]. The most important optical techniques are infrared (IR) and Raman spectroscopy; both of which are well established methods for studying sample types such as biofluids, tissues and cell cultures.

Raman scattering, or inelastic scattering, is a low probability event (1 in 108) that occurs when a sample is exposed to a monochromatic laser light source and undergoes molecular polarizability changes [12,13,14]. A shift in the frequency of the incident light occurs as molecules in the sample are excited, emitting photons to provide a quantifiable signal, without causing any damage to the sample. As the signal intensity of the scattered light is proportional to the concentration of a molecule within the sample, the overall Raman spectrum generated from an unknown sample can provide information on both its molecular constituents and the concentration of these present in the sample [15]. In summary, this rapid, label-free and cost-effective technique can provide a surrogate read-out to describe the metabolomic profile of a patient sample and has been successfully used across a wide area of clinical medicine, including complex disorders, such as Alzheimer’s disease [16,17], multiple sclerosis [18], primary immune deficiency [19], autoantibody-associated vasculitis [20,21], HIV/AIDS [22], diabetes [23] and carcinogenesis [24,25,26,27,28]. High diagnostic accuracy has been demonstrated for classification of numerous cancer types and other biological applications [29,30,31,32,33,34].

Raman spectroscopy is a candidate to overcome the shortfalls with current laboratory methods and improve the diagnostic pathway for SLE. To date, Raman has limited applications in autoimmunity [35,36,37] and remains a novel tool in the assessment of serum anti-dsDNA antibody profiles in SLE patients. In our proof-of-concept study, we explore the use of Raman spectroscopy to classify serum samples from SLE patients and healthy controls. Our initial findings are encouraging for the development of a future diagnostic test and could provide an important contribution to delineate the anti-dsDNA antibodies profile in SLE patients.

## 2. Materials and Methods

### 2.1. Raman Spectroscopy Study

#### Sample Collection and Preparation

A total of 234 Raman spectra were examined (20 replicates per participant dependent on sample volume) to investigate key variances between SLE patients (*n* = 8) and HC (*n* = 4). As such, there were 154 SLE Raman spectra and 80 HC Raman spectra collected in total. Surplus serum samples from eight SLE patients received at LTHTR Clinical Immunology laboratory for routine testing were saved for Raman spectroscopy. Each of these patients had been previously diagnosed with SLE, according to classification criteria [1,2]; therefore, samples were not taken at the timepoint of initial diagnosis. Each sample represented a different individual patient. Longitudinal testing was not undertaken as a part of this study. Samples were anonymised and collated into subgroups 1–3 based on their dsDNA antibody test results; 1—ELIA Negative (Neg), CLIFT not performed (N/A) (*n* = 2); 2—ELIA Positive (Pos), CLIFT Neg (*n* = 2); 3—ELIA Pos, CLIFT Pos (*n* = 4) (Table 1). All SLE patients were positive for anti-nuclear antibodies (ANA) by indirect immunofluorescence testing on Hep-2 slides (Innova). To ensure double-blind analysis, a random number generator was used to assign sample study IDs. Samples were gathered within 1 week of blood collection, aliquoted into anonymised tubes and stored at −80 °C until processing. Control samples consisted of four serum samples collected from healthy laboratory staff volunteers, obtained following informed consent. Samples were anonymised from the time of collection, assigned and labelled with only a unique random identification number. A record of ID numbers used for healthy controls was documented with SLE patient samples for future reference, omitting volunteer identifiers. All samples were stored at −80 °C until analysed, first thawing at room temperature.

### 2.2. Ethics Statement

The Health Research Authority Research Tool was used in the planning stages, which determined that this project was not a piece of research that required ethical approval. This project proposal was reviewed by the Centre for Health Research and Innovation on behalf of Lancashire Teaching Hospitals NHS Trust (LTHTR) and was considered a service evaluation not requiring ethical or governance review.

### 2.3. Spectral Acquisition

Samples were processed using Thermo Scientific^TM^ DXR^TM^3 dispersive Raman Microscope aligned and calibrated for 532 nm laser wavelength using automated techniques, as per the OMNIC user protocol. Optimal Raman parameters (532 nm laser wavelength, 10 mW laser power, ×10 objective, 20 s exposure time, 10 exposures, 512 background exposures, 900 lines/mm grating, 50 µm pinhole spectrograph aperture, 1 µm spatial resolution and 2 µm confocal depth) were selected and were inputted into OMNIC software prior to spectra collection. An amount of 50 µL of serum was pipetted onto a 75 × 25 × 1 mm calcium fluoride (CaF_2_) slide (Knight Optical). The microscope (1 micron sampling area) was navigated around different parts of the homogenous sample to collect 10 different spectral points. A total of 10 spectra were collected within an approximate 30 min before the CaF_2_ slide was cleaned with alcohol disinfectant wipes (Medipal) and diH_2_0 and dried. A further 10 point spectra was subsequently collected with a fresh 50 µL of sample pipetted onto the CaF_2_ slide. Each sample was processed, as per this process, one at a time collecting in total 20 spectra per sample consecutively within 1 h. This resulted in a total acquisition of 234 Raman spectra, 154 spectra collected from 8 individual SLE patients and 80 spectra collected from 4 HCs. For 3 of the SLE patients it was not possible to acquire the full 20 replicates; a minimum number of 17 replicates were collected for each sample. Samples were processed in a double-blinded manner, only identifiable by their unique random ID. All samples were processed over six days.

### 2.4. Spectral Pre-Processing

Analysis of the spectral datasets was performed using the IRootLab toolbox (trevisanj.github.io/irootlab/; accessed on 6 April 2022), within MATLAB R2017a software (MathWorks, Natick, MA, USA), unless stated otherwise. Pre-processing consisted of rubber-band-like baseline correction and vector normalisation performed on raw spectral data.

### 2.5. Multivariate Analysis and Model Validation

As a means of supervised multivariate analysis, principal component analysis linear discriminant analysis (PCA-LDA) was used as a classifier [38]. In addition to PCA-LDA, supervised classification was also performed by partial least squares discriminant analysis (PLS-DA), which is a classification technique based on a partial least squares (PLS) model applied to the pre-processed data, reducing them to a few numbers of latent variables (LVs), followed by a discriminant analysis classifier [39]. PLS-DA maximises the co-variance between the spectral data and the sample category, where the samples are assigned to classes based on a straight line that divides the classes’ space [39].

Classification was performed by measuring the PCA-LDA scores and by the predicted response of the PLS-DA model. PCA-LDA scores ($L_{ik}$) are calculated based on the following equation:

(1) $$L_{ik}={\left(x_{i}-{\bar{x}}_{k}\right)}^{T}\Sigma_{\mathrm{pooled}}^{-1}\left(x_{i}-{\bar{x}}_{k}\right)-2\log_{e}\pi_{k}$$

where $x_{i}$ are the PCA scores for sample i; ${\bar{x}}_{k}$ is the mean PCA scores for class k; $\Sigma_{\mathrm{pooled}}$ is the pooled covariance matrix; and $\pi_{k}$ is the prior probability of class k [38].

The PLS-DA predicted response ($\hat{y}$) is calculated based on Equation (2):

(2) $$\hat{y}=Xb$$

where X is the pre-processed spectral data and b is a regression vector calculated through a series of iterations using both spectral and class category information [40].

Relevant biomarker peaks were found using a cluster vector approach [41], which is a method based on PCA that creates a “loadings-like” plot for the three PCs whose projections give the best cluster separation. This is performed by the sum of the three loading vectors weighted by the median scores, and the resultant vector (cluster vector) shows the weight for the most important wavenumbers responsible for class separation [41]. Additional statistical tests were performed on the PCA-LDA scores and on the absorbance intensities for the main peaks identified by the cluster vector approach based on ANOVA, where *p*-values were calculated for statistical significance at a 95% confidence level (*p* < 0.05).

A boxplot was generated for the PCA-LDA scores to facilitate the visualisation of class separation.

Finally, the models were validated by Monte Carlo cross-validation performed with 1000 iterations and leaving 20% of samples out for validation. Monte Carlo is an exhaustive type of cross-validation technique that performs a great number of iterations where, for each iteration, 20% of the data are randomly left out for validation; thus, the classification model is built with 80% of the data and predicted on the remaining 20% [42]. At the end, the mean accuracy, sensitivity and specificity were reported for each model, as well as the mean predicted response displayed in a form of confusion table. The accuracy (AC), sensitivity (SENS) and specificity (SPEC) were calculated for each class as follows:

(3) $$\mathrm{AC}\left(\%\right)=\left(\frac{\mathrm{TP}+\mathrm{TN}}{\mathrm{TP}+\mathrm{FP}+\mathrm{TN}+\mathrm{FN}}\right)\times 100$$

(4) $$\mathrm{SENS}\left(\%\right)=\left(\frac{\mathrm{TP}}{\mathrm{TP}+\mathrm{FN}}\right)\times 100$$

(5) $$\mathrm{SPEC}\left(\%\right)=\left(\frac{\mathrm{TN}}{\mathrm{TN}+\mathrm{FP}}\right)\times 100$$

where TP stands for true positive, TN for true negative, FP for false positive and FN for false negative [43].

### 2.6. Retrospective Clinical Audit of Anti-dsDNA Antibody Results in SLE Patients

A retrospective clinical audit was performed on 600 anti-dsDNA test requests over an 18-month period. Anti-dsDNA antibody results from enzyme-linked immunoassay (EliA; Phadia/Thermo Fisher Scientific, Waltham, MA, USA), and *Crithidia luciliae* immunofluorescence testing (CLIFT; Euroimmun, Lübeck, Germany) was gathered and analysed to determine clinical sensitivities and specificities for the local population, including statistical analysis (SPSS). Results of requests from primary and secondary care services at St Helens and Knowsley Teaching Hospitals NHS Trust (STHK) and Southport and Ormskirk NHS Trust were gathered. Only requests that had a positive connective tissue disease (CTD) screen and/or a positive anti-nuclear antibody (ANA) result by HEp-2 IIF were included. CTD screen and Hep-2 testing were performed by the Immunology department at Whiston Hospital (St Helens and Knowsley Teaching Hospitals NHS Trust, Prescot, UK); anti-dsDNA antibodies were performed by Lancashire & Lakeland Immunology Service at Royal Preston Hospital (Lancashire Teaching Hospitals NHS Foundation Trust). Patient identifiers were anonymised once all data were gathered prior to analysis.

### 2.7. Detection of Anti-dsDNA Antibodies

Samples included in the audit had ANA, including autoantibodies to U1RNP, SS-A/Ro, SS-B/La, Centromere B, Scl-70, Jo-1, Fibrillarin, RNA Pol III, Rib-P, PM-Scl, PCNA, Mi-2, SmD, and dsDNA, tested by STHK immunology using ELIA connective tissue disease screen (ThermoFisher Phadia250) and HEp-2 (Innova, Rome, Italy) IIF. Positive samples were confirmed at LTHTR immunology by the same methods. Anti-dsDNA antibodies were analysed by ELIA (ThermoFisher Phadia 250) at Preston immunology. Positive ELIA dsDNA results were confirmed by IIF-detecting antibodies to *Crithidia luciliae* (Euroimmun).

### 2.8. Data Analysis

Data were exported from laboratory information management systems to Microsoft Excel for analysis. Data comparisons were made using Microsoft Excel line, scatter and bar graphs. SPSS (IBM) was used to compare datasets of anti-dsDNA antibody methods by ELIA and CLIFT. Negative, weak positive, positive and strong positive CLIFT interpretations were assigned values of 0, 1, 2 and 3, respectively, and were compared with the ELIA quantitation on the same sample.

Association between the 4 CLIFT interpretations and respective ELIA dsDNA results were analysed using a Kruskal–Wallis test in SPSS. Associations of two CLIFT groups in SLE patients at diagnosis were made with Mann–Whitney U Test in SPSS.

## 3. Results

### 3.1. PCA and PCA-LDA Clustering of Raman Spectra for Discrimination of SLE Patients from Healthy Controls

The major aim of this proof-of-concept study was the discrimination of SLE patients from HC in blood serum using Raman spectroscopy and multivariate analysis techniques.

**Figure 1:**
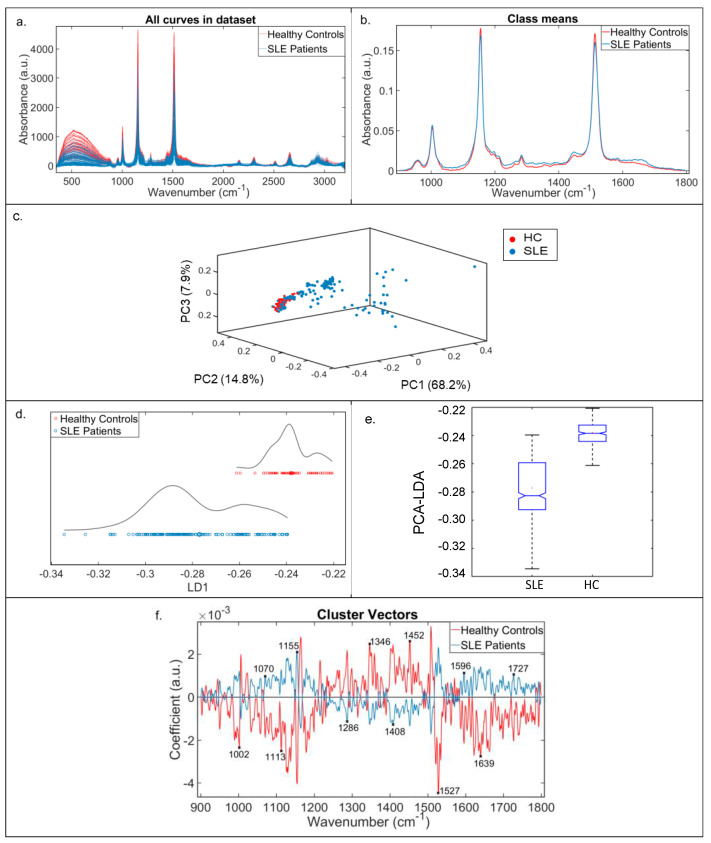
Raman spectral data for SLE patients (SLE) vs. healthy controls (HC) for serum samples. (**a**) All raw spectra curves of healthy controls (red) and SLE patients (blue). Whole region of wavenumbers (400–3500 cm^−1^) against the absorbance intensity (a.u). (**b**) Average rubber band, vector normalised pre-processed Raman spectra cut to biological fingerprint region (900–1800 cm^−1^). (**c**) The 3D PCA scatterplot. Pre-processed Raman data using PC1, PC2 and PC3. The 3D scatterplot shows separation of healthy control (red) and SLE (blue). (**d**) PCA-LDA discriminant scores. Score plots show clear separation of groups, healthy controls (red) and SLE patients (blue) (*p*-value < 0.0001). (**e**) Box plot for healthy controls (HC) and SLE patients. Mean and IQRs illustrated in boxes; SDs displayed in whiskers. (**f**) PCA-LDA cluster vector analysis. Cross-validated cluster vector analysis demonstrates discriminating peaks between healthy controls (red) and SLE patients (blue). The top 12 discriminating peaks are labelled with their wavenumber. IQR—Interquartile range; SD—Standard deviation.

Initial analysis examined spectra (20 replicates per participant dependent on sample volume) for SLE patients (*n* = 8) versus HC (*n* = 4). As such, there were 154 SLE spectra collected in total and 80 HC spectra. The total raw spectra (400–2500 cm^−1^) and average pre-processed spectra cut to the fingerprint region (900–1800 cm^−1^) are shown in Figure 1a,b, respectively. Rubber band baseline correction and vector normalisation produces spectra for the crude visualisation of differences between the two groups. This recognised technique corrects for experimental variation and improves the accuracy and interpretability of the data whilst maintaining spectral integrity. As expected, there was a high degree of overlap between the serum biofluid spectra generated for SLE patients and HCs, with prominent signatures associated with proteins and lipids across the fingerprint region [44].

To identify the more subtle, important discriminatory spectral signatures between disease groups, multivariate analysis and machine learning techniques must be subsequently applied to pre-processed spectra. For the further interrogation of variance between the two classes, an exploratory (unsupervised) analysis using PCA was undertaken, followed by a supervised method of class separation, PCA-LDA, to enable successful segregation of subjects into their respective groups. The 3D PCA scatterplot in Figure 1c shows a reasonable separation of the SLE spectra from HC spectra across PC1, PC2 and PC3. Superior class separation was achieved using PCA-LDA; scores plot in Figure 1d and box plot in Figure 1e illustrate clear class separation and illustrate significant differences between the SLE patient spectra and HCs (*p* < 0.0001). *p*-values calculated based on an ANOVA test. In the SLE patient group, the mean PCA-LDA score was lower compared to the HC group, with a larger spread of data observed within the SLE patient cohort (larger interquartile range and standard deviation), compared to the HCs (Figure 1e). This would be in keeping with the high degree of clinical and serological heterogeneity reported in SLE.

### 3.2. Key Discriminating Wavenumbers between SLE Patients and HC

**Figure 2:**
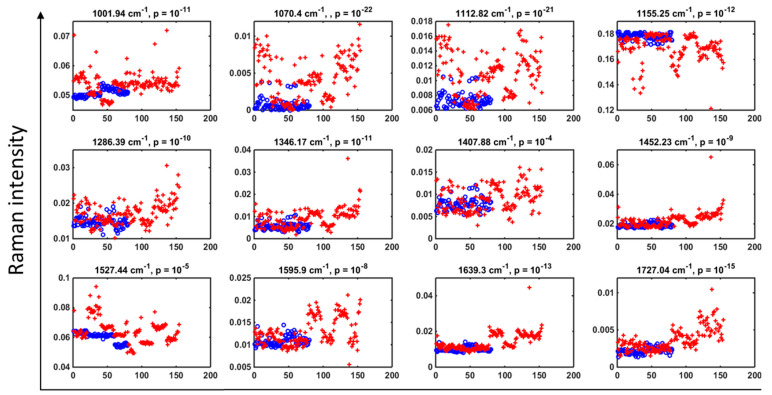
PCA-LDA discriminant scores for SLE-associated biomarkers. Raman intensity for each biomarker peak along with their *p*-value: healthy controls (o) and SLE patients (+). All peaks were found to be highly statistically significant at *p* < 0.001. *p*-value calculated based on an ANOVA test.

Cross-validated PCA-LDA cluster vectors were generated to identify the 12 most discriminatory peaks between the two classes (Figure 1f). The wavenumbers responsible for class separation were: 1002 cm^−1^, 1070 cm^−1^, 1113 cm^−1^, 1155 cm^−1^, 1286 cm^−1^, 1346 cm^−1^, 1408 cm^−1^, 1452 cm^−1^, 1527 cm^−1^, 1596 cm^−1^, 1639 cm^−1^ and 1727 cm^−1^. Tentative molecular assignments (Table 2) have been attributed to each wavenumber, obtained from a literature review of Raman research studies performed in biological tissues [45], and from source data embedded within the Matlab toolbox ‘irootlab’ [45]. The comparison of Raman peak intensities between SLE patients and HC were found to be highly statistically significant at *p* < 0.001 for all 12 wavenumbers, calculated based on an ANOVA test. Significant increases in Raman intensity were demonstrated in 11 of the 12 discriminating wavenumbers for SLE patients, compared to HCs, with a single peak at 1155 cm^−1^ demonstrating reduced Raman intensity within the SLE group (Figure 2).

### 3.3. SLE Patients Successfully Segregate from HC Using PCA-LDA and PLS-DA Classification Models

Before model construction, 234 spectra (pre-processed; rubber band baseline correction and vector normalisation) were assigned to the training set (80% of spectra), and the validation set was generated based on a Monte Carlo cross-validation algorithm containing 20% of spectra randomly selected during 1000 iterations. The PCA-LDA model was built with 10 PCs and the PLS-DA model built with six LVs. The training set was used for model construction and the validation set for final model evaluation. Performance characteristics (accuracy, sensitivity and specificity) were calculated based on the ability of the model to correctly classify spectra in the test dataset. The accuracy represents the total number of spectra correctly classified considering true and false negatives, the sensitivity represents the portion of positives correctly classified, and the specificity represents the portion of negatives correctly classified [64].

**Figure 3:**
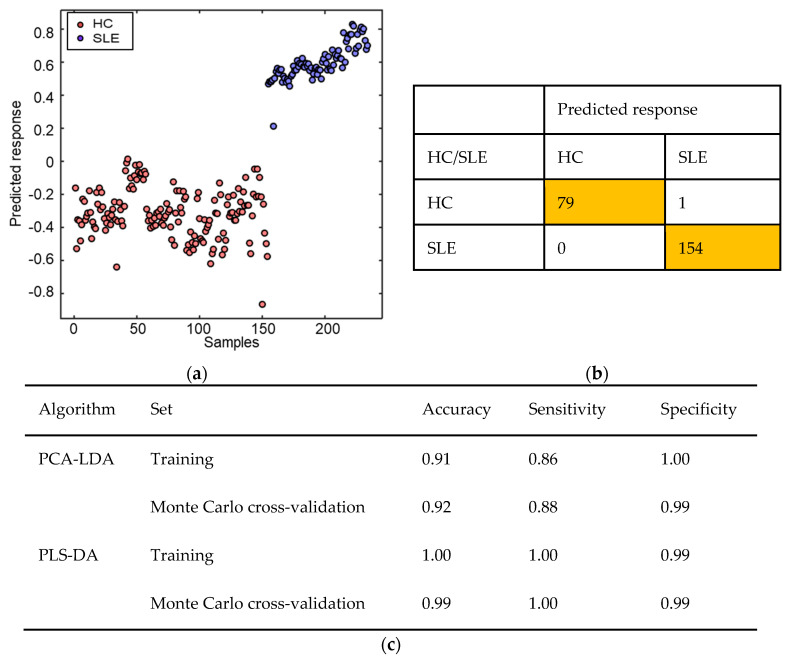
PCA-LDA and PLS-DA results for classification of SLE patients and HC using Raman serum analysis. (**a**) PLS-DA classification model predicted response for SLE patients (red circles represent 154 individual spectra for 8 patients) and HC (blue circles represent individual spectra for 4 participants). (**b**) Cross-validation confusion table generated from individual spectra, 154 SLE spectra (*n* = 8 patients) and 80 HC spectra (*n* = 4 participants). (**c**) Classification model performance characteristics. Note: PCA-LDA model built with 10 PCs, and PLS-DA model built with 6 LVs. Cross-validation: Monte Carlo leaving 20% of samples out with 1000 interactions. Pre-processing: rubber band baseline correction and vector normalisation.

The predicted response based on the constructed PLS-DA classification model illustrates outstanding segregation between the SLE patients and HC, Figure 3a. Of the total 154 SLE spectra and 80 HC spectra, there was a single spectrum in the HC group that was incorrectly classified (Figure 3b) and shown in Figure 3a as a single blue circle between the two group clusters. Figure 3c illustrates the model performance of the algorithms evaluated. Superior results were obtained from the PLS-DA model, with 99% accuracy, 100% sensitivity and 99% specificity. These metrics demonstrate an outstanding classification rate for distinguishing between the two groups. The performance of the PCA-LDA model also demonstrates excellent results with 92% accuracy, 88% sensitivity and 99% specificity.

### 3.4. PCA and PCA-LDA Clustering of Raman Spectra from Three SLE Subgroups and HCs

**Figure 4:**
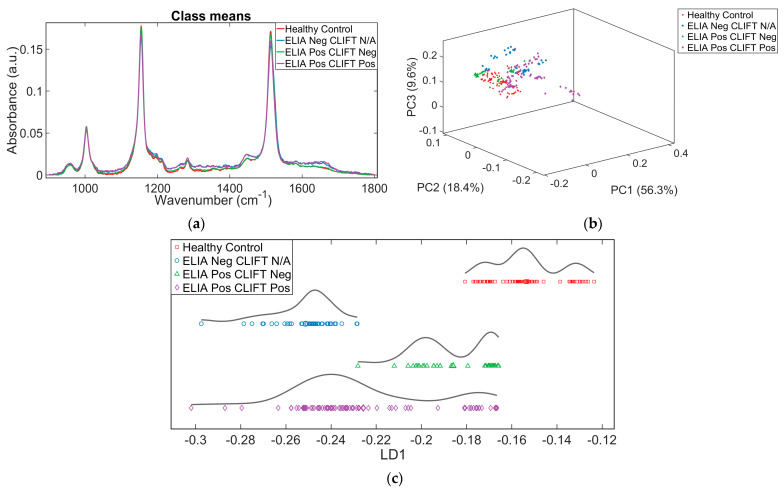
Raman data for SLE subgroups and HCs. SLE subgroups based on serological laboratory results (ELIA and CLIFT). ELIA Neg CLIFT N/A (*n* = 2; 40 individual spectra); ELIA Pos CLIFT Neg (*n* = 2; 40 individual spectra); ELIA Pos CLIFT Pos (*n* = 4; 80 individual spectra); HC (*n* = 4; 80 individual spectra). (**a**) Average pre-processed (Rubber band baseline correction, vector normalisation) Raman spectra cut to biological fingerprint region (900–1800 cm^−1^). (**b**) The 3D PCA scatterplot. Pre-processed Raman data shows separation between groups using PC1, PC2 and PC3; HC—red, and SLE subgroups, ELIA Neg CLIFT N/A—blue, ELIA Pos CLIFT Neg—green, ELIA Pos CLIFT Pos—purple. (**c**) The 1D PCA-LDA scatterplot. PCA-LDA discriminant scores plot shows clear separation between the groups (*p*-value < 0.001). HC—red, and SLE subgroups, ELIA Neg CLIFT N/A—blue, ELIA Pos CLIFT Neg—green, ELIA Pos CLIFT Pos—purple. *p*-value calculated based on a MANOVA test (*p* = 4.90 × 10^−4^).

The eight SLE patient serum samples were further allocated into three subgroups based on the results from antibody testing (ELIA dsDNA and CLIFT), ELIA Neg CLIFT N/A (*n* = 2), ELIA Pos CLIFT Neg (*n* = 2) and ELIA Pos CLIFT Pos (*n* = 4). Raman spectra of serum samples in each group was analysed alongside HCs (*n* = 4). Crude visualisation of the pre-processed (rubber band baseline corrected, vector normalised) spectra illustrates a large overlap between the spectral signatures of each group as expected, and as previously seen for the Raman spectra of total SLE patients and HCs (Figure 4a). PCA analysis illustrates some clustering and reasonable separation between the subgroups (Figure 4b); however, as PCA is an unsupervised technique it does not have the power to clearly segregate the spectra into their respective groups. Subsequent PCA-LDA clearly demonstrates class separation (Figure 4c), with the discriminant scores calculated to show significant variation between the four groups (*p*-value < 0.001) based on a MANOVA test.

### 3.5. SLE Patient Subgroups and HC Successfully Segregate Using PCA-LDA and PLS-DA Classification Models

PCA-LDA and PLS-DA were applied to build classification models based on the subgroups of patients. The training set was used for model construction and the validation set for final model evaluation. Performance characteristics (accuracy, sensitivity, and specificity) were calculated based on the ability of the model to correctly classify spectra in the test dataset. The accuracy represents the total number of spectra correctly classified considering true and false negatives; the sensitivity represents the portion of positives correctly classified into their respective classes, either the HC or SLE subgroups, ELIA Neg CLIFT N/A (CLIFT N/A), ELIA Pos CLIFT Neg (CLIFT Neg), or ELIA Pos CLIFT Pos (CLIFT Pos); and the specificity represents the portion of negatives correctly classified. Before model construction, 234 spectra (pre-processed by rubber band baseline correction and vector normalisation) were assigned to the training set (80% of spectra), and the validation set was generated based on a Monte Carlo cross-validation algorithm containing 20% of spectra randomly selected during 1000 iterations. The PCA-LDA model was built with 10 PCs and the PLS-DA model built with eight LVs.

**Figure 5:**
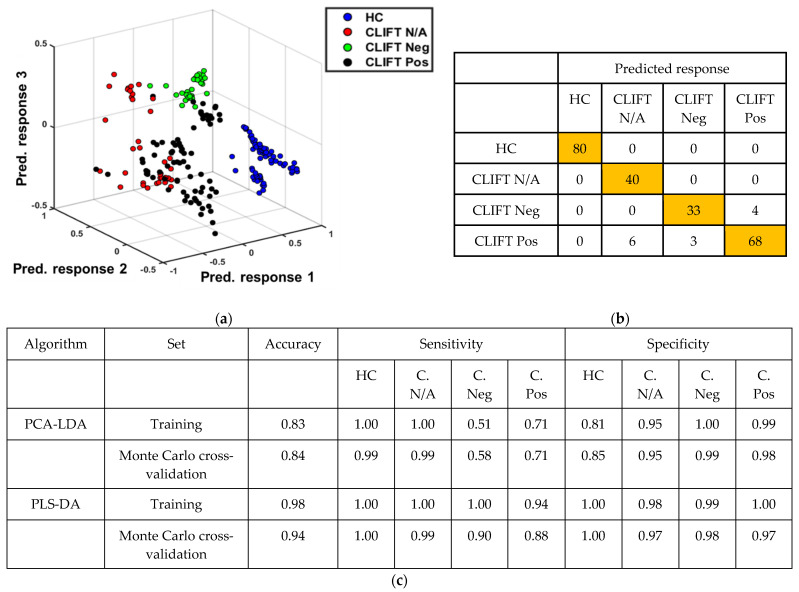
Multivariate analysis of the fingerprint region for SLE subgroups and HCs. (**a**) PLS-DA classification model predicted response for healthy controls (HC), ELIA Neg CLIFT N/A (CLIFT N/A), ELIA Pos CLIFT Neg (CLIFT Neg), ELIA Pos CLIFT Pos (CLIFT Pos). (**b**) Cross-validation confusion table generated from individual spectra; 154 SLE spectra (*n* = 8 patients) and 80 HC spectra (*n* = 4 participants). (**c**) Classification model performance characteristics. Note: PCA-LDA model built with 10 PCs, and PLS-DA model built with 8 LVs. Cross-validation: Monte Carlo leaving 20% of samples out with 1000 interactions; C.—CLIFT. Pre-processing: rubber band baseline correction and vector normalisation.

The predicted response based on the constructed PLS-DA classification model again demonstrates outstanding segregation between the SLE subgroup patients and HC, Figure 5a. Between the SLE subgroups, the separation is not as strong, with some overlap illustrated between the groups, particularly for the CLIFT N/A patients and CLIFT Pos patients. However, there is clear clustering of the spectra observed in their respective subgroups, with the CLIFT Neg patients forming the most discrete cluster. Of the total 154 SLE spectra and 80 HC spectra, there were 13 spectra that were incorrectly classified (Figure 5b); the incorrect predicted responses mostly affect the CLIFT Pos spectra, shown as black dots in Figure 5a. Figure 5c illustrates the model performance of the algorithms tested. Again, superior results were obtained from the PLS-DA model, with 94% accuracy, 94% average sensitivity and 98% average specificity. These metrics demonstrate an outstanding classification rate for distinguishing between the four groups. The performance of the PCA-LDA model also demonstrates highly commendable results with 84% accuracy, 78% average sensitivity and 94% average specificity.

### 3.6. Retrospective Clinical Audit of Anti-dsDNA Antibody Results in SLE Patients

#### 3.6.1. Individual Sample Relationship between Results by ELIA dsDNA and CLIFT

**Figure 6:**
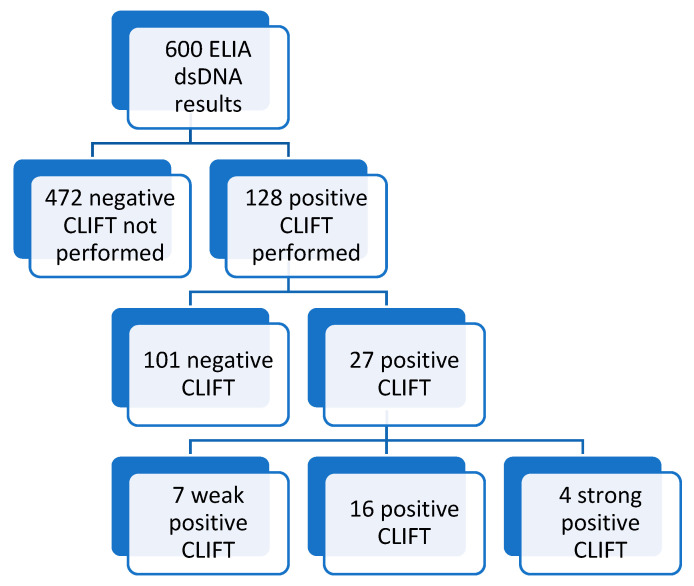
Summary of results from audit of 600 sample requests for anti-dsDNA antibody testing.

A retrospective clinical audit was performed on 600 anti-dsDNA test requests over an 18-month period. A total of 128 (21%) were positive for dsDNA antibodies by ELIA methodology. As per the local laboratory testing protocol, all ELIA dsDNA positive results (>10 IU/mL) were subsequently tested by IIF CLIFT methodology. Of the 128 with CLIFT performed, 101 (79%) were negative and 27 (21%) were positive. Of the 27 samples positive by CLIFT, the interpretations included 7 weak positive (26%), 16 positive (59%) and 4 strong positive (15%). Therefore, of the total 600 requests for anti-dsDNA antibodies, only 27 samples (5%) were positive for anti-dsDNA antibodies by both ELIA and CLIFT (Figure 6).

**Figure 7:**
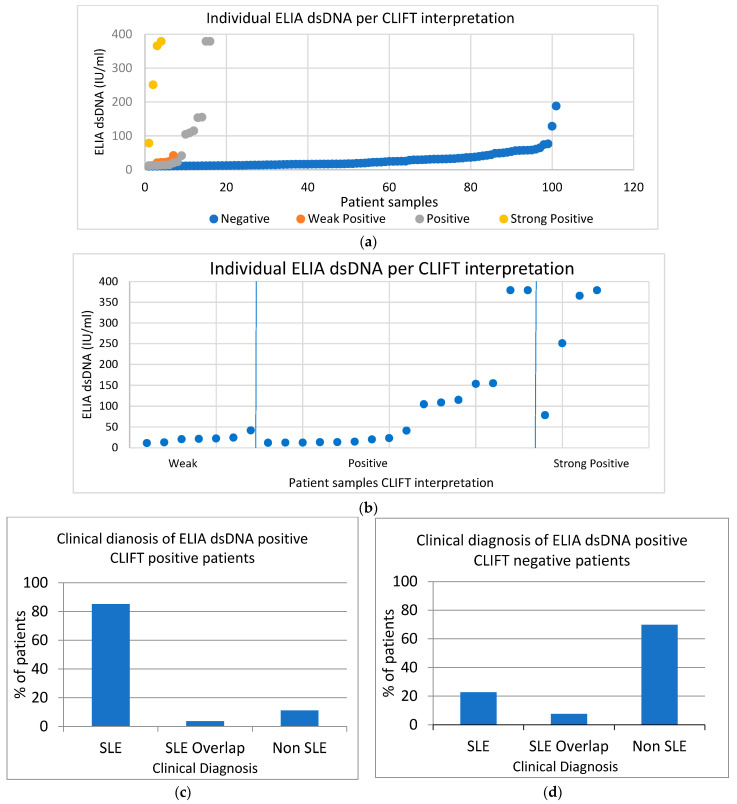
Relationship between quantitative ELIA dsDNA and CLIFT interpretations. (**a**) All ELIA dsDNA values of individual samples grouped into their CLIFT interpretation groups. Groups consist of negative (blue circle), weak positive (orange circle), positive (grey circle) and strong positive (yellow circle). (**b**) Separations of weak positive, positive and strong positive CLIFT interpretations. Trend of ELIA dsDNA value increases as interpretation increases from weak positive to strong positive. (**c**) Clinical diagnosis of patients with positive anti-dsDNA antibody results by ELIA and confirmed positive by CLIFT. A total of 85% of patients had an SLE diagnosis. A total of 4% had an SLE overlap syndrome. A total of 11% of patients had other diagnoses, excluding SLE. (**d**) Clinical diagnosis of patients positive for anti-dsDNA antibodies by ELIA but negative by CLIFT. A total of 23% of patients had an SLE diagnosis. A total of 7.5% of patients had SLE overlap syndromes. A total of 70% of patients did not have an SLE diagnosis.

Figure 7a,b include data from all samples that were ELIA dsDNA positive (128) and their accompanying CLIFT interpretation, regardless of diagnosis. The majority of the CLIFT-negative group (*n* = 101) had ELIA dsDNA values focused towards the lower end of positive (10 IU/mL); however, there were outliers, which reached up towards 200 IU/mL. Positive CLIFT results (*n* = 27) appear in three distinct groups. One group was towards the lower end of ELIA values, similar to CLIFT-negative samples. The weak positive CLIFTs also sit within this population. A second cluster or CLIFT-positive samples appears mid-range of positive ELIA values between 100–170 IU/mL. A further cluster of positive CLIFT samples have the highest ELIA values at >379 IU/mL. Strong positive CLIFT samples generally have higher-end ELIA values, greater than 250 IU/mL with one slightly lower at 70 IU/mL. These findings indicate that whilst at the ‘negative’, ’weak positive’, and ‘strong positive’ CLIFT interpretation there appears to be a relationship between quantitative ELIA values, there is poor correlation between quantitative ELIA values in the CLIFT ‘positive’ group.

#### 3.6.2. Clinical Diagnoses of Patients with Positive Anti-dsDNA Antibody Results

Figure 7c,d show the clinical diagnosis of patients with a range of anti-dsDNA antibody results. In Figure 7c, the greatest number of SLE diagnoses were seen in patients positive for anti-dsDNA antibodies by both ELIA and CLIFT, which accounted for 85% of results. However, 11% of patients with dual positive (ELIA and CLIFT) did not have SLE. In Figure 7d, 23% of patients with a single positive dsDNA result (by ELIA only) were diagnosed with SLE. In summary, these results highlight the high clinical utility of dsDNA antibodies in the diagnosis of SLE, particularly in patients with dual positivity by ELIA and CLIFT. However, our findings highlight the need for an improved diagnostic pathway, given that 11% of dual positive patients did not have SLE, and 23% of patients with a single positive by ELIA and negative CLIFT had a clinical diagnosis of SLE.

#### 3.6.3. Sensitivity, Specificity, Positive Predictive Value and Negative Predictive Value

Our audit confirmed the SLE testing pathway used locally was in keeping with recommendations described in the guidelines, i.e., the presence of anti-dsDNA antibodies were confirmed with a high specificity assay [8]. Our first line screening by ELIA dsDNA is the more sensitive method with 81.0%, compared to CLIFT sensitivities of 67.7% (Table 3). The confirmatory CLIFT was the more specific method with a 95.7% specificity, compared to ELIA’s 83.7%. The positive predictive value (PPV) was higher for CLIFT than ELIA with 85.2% and 24.8%, respectively, whereas ELIA had a greater negative predictive value (NPV) at 98.5%, compared to CLIFT’s 89.1%.

## 4. Discussion

Using blood-based vibrational spectroscopy, we achieved results with significant clinical relevance in the classification of patients with SLE from healthy controls (HC). We also demonstrate that Raman spectroscopy could detect differences not only between SLE patients and HCs but also between SLE subgroups categorised using combinations of serological results (dsDNA antibodies) obtained from two different laboratory methods (ELIA and CLIFT). We achieved sensitivities and specificities of 100% and 99%, respectively, for the segregation of SLE patients from HCs, and average sensitivities and specificities of 94% and 98% for successful subgroup classification. These proof-of-concept findings highlight the potential of Raman spectroscopy as an inexpensive tool for screening, diagnosis and management of SLE.

Antibodies to SLE are clinically important and have been used historically in the diagnosis and classification criteria for SLE [1]. The diagnostic criteria have broadened from the early days of diagnosis, reflecting the complexity and clinical heterogeneity of the disease. There are several SLE-associated antibodies, which have variable sensitivity and specificity for SLE. Antibodies to double-stranded DNA (dsDNA) are considered a highly specific (97.4%) marker for SLE and have a high frequency in disease (70–98%) [65,66]. Although considered virtually diagnostic, known problems with the assays used to detect them are still evident and doubts have been raised about their significance in terms of disease pathogenesis. Nevertheless, these tests remain widely used in the diagnosis, and monitoring of SLE patients as an alternative testing platform has yet to become routinely available.

In relation to problems with the assays, it is known across immunology laboratories that the results for the detection of dsDNA from the two routinely used methodologies (ELIA and CLIFT) are not always well correlated. We confirmed this finding in our retrospective audit, where only 21% of samples positive by ELIA were also positive by CLIFT, of which 85% had a diagnosis of SLE. There is an unmet need for new laboratory methods in the diagnosis and monitoring of SLE patients.

SLE is a multifactorial autoimmune disease with a wide range of clinical manifestations and severity. There is also variability across serological test results and there remains a diagnostic delay of around 6.4 years, highlighting a need for improvement. Novel technology could be the solution to improving the diagnostic testing for SLE, with Raman spectroscopy being an inexpensive, well-placed methodology for the investigation of pathological disease. Raman spectroscopy is a mode of vibrational spectroscopy that can provide molecular-level information on all the biochemical components within a sample. Spectral bands are molecule-specific; therefore, the unique spectrum generated allows the investigation of functional groups, bonding types and molecular conformations [67]. As the biochemical constituents of a sample will be influenced and altered by both health and disease, Raman spectroscopy is a well-placed candidate for the investigation of pathological samples.

We found when examining the Raman spectra of all SLE patients (determined clinically, independent of serology results for dsDNA) that there was outstanding segregation between SLE patients and HC. The PLS-DA classification model demonstrated 99% accuracy, 100% sensitivity and 99% specificity, illustrating excellent correlation between spectral features and SLE. The comparison of SLE and HC spectra following PCA-LDA cluster vector analysis enabled the identification discriminatory peaks that clearly differentiate between the two groups. The 12 most discriminatory peaks identified with high statistical significance could serve as a panel of spectral markers indicative of disease. Using both the PCA-LDA and PLS-DA chemometric techniques to predict response (SLE patient or HC), there was a clear and significant (*p* value <0.0001) segregation between the two groups. This indicates that the Raman spectra are significantly different between disease group and controls, reflecting potential contributions of numerous disease-specific biomarkers present in the serum. The evidence of within-group clustering was clear within the HCs, and, albeit with a larger variance, also present within the SLE patient group. This finding was in line with our expectations, based on the known clinical heterogeneity of SLE patients, and anticipated biochemical changes that could occur within the serum during active disease states, compared to patients with clinically stable disease.

The aim of including subgroups of SLE patients defined by the results of the current serological test was to examine whether Raman spectroscopy would similarly classify them into discrete groups based on the total composition of biochemical components present in the serum. If so, this would suggest that additional disease-specific biomarkers, not just dsDNA antibodies, are responsible for the difference between the subgroups, or alternatively, that the dsDNA antibodies in the serum are chemically or structurally altered in the subgroups, resulting in spectral variations between the groups.

Of interest, when focusing on a single serological biomarker (dsDNA antibodies) and subgrouping patients based on results obtained using two methods (negative by ELIA/CLIFT, positive ELIA/negative CLIFT, and positive by both ELIA and CLIFT), the segregation between SLE and HC spectra was not as clear. We observed overlapping predicted responses in the PCA-LDA and PLS-DA classification models across the three SLE subgroups, particularly evident in patients with a positive dsDNA antibody result obtained by either ELIA alone, or by both ELIA and CLIFT together. This indicates that the variable results obtained from dsDNA antibody testing methods did not correlate as strongly with specific Raman spectral features. We observed reasonable clustering into the discrete subgroups, but to a lesser degree than in the total SLE vs. HC analysis.

These findings, demonstrated by the lower accuracy, sensitivities and specificities achieved within the PCA-LDA and PLS-DA classification models, indicate that there is not the same clear correlation between the Raman spectra of SLE sub-group patients when incorporating the dsDNA test results. As such, there must be variation in the assays that detect the dsDNA antibodies, and it is important that the test platforms are not all capable of detecting the same antibodies, i.e., are unable to detect biochemically or structurally altered antibodies. To improve the current testing pathway for SLE patients, we require a clear-cut, unequivocal means to identify SLE patients, and successfully segregate them from HCs. The investigation of serum using Raman spectral features and classification models may provide this improved diagnostic pathway. 

To interpret Raman spectral differences in biomedical studies and assign molecular associations, which may contribute to disease-specific changes, researchers can use published literature databases and libraries. Based on the molecular assignments attributed within the irootlab toolbox [45], the 12 most significant spectral bands identified to contribute to the discrimination between SLE patients and HC were associated with protein phosphorylation, a form of post-translational modification (PTM). We observed an increased Raman intensity in 11 of the 12 peaks within the SLE patients group, which may signify an increased rate of protein phosphorylation occurring in SLE patients, compared to HCs.

Proteins are synthesised by ribosomes through the translation of mRNA, most of which subsequently undergo a modification known as PTM. The changes include physical and chemical changes, which have an influence on the functional diversity, stability, and molecular interactions of the protein. The common forms of PTM are trimming or proteolysis, ubiquitination, and covalent modifications (i.e., phosphorylation, acetylation, hydroxylation, and methylation). Other mechanisms of PTM include the addition of a complex molecule (i.e., glycosylation), or the modification of amino acids (i.e., deamidation and citrullination) [68]. PTMs can occur in both health and disease.

Under conditions of inflammation and cellular stress, the formation of reactive oxygen species and the induction of enzymes can lead to an increase in the formation of PTMs [69]. These modifications are implicated in human diseases, such as autoimmunity, and occur when the proteins our immune system previously classified as ‘self’ are recognised as new ‘non-self’ proteins. This leads to a breakdown of tolerance, and the generation of an autoimmune response within the body. The PTM, citrullination is widely implicated in disease, as autoimmune responses against citrullinated proteins are generated. These disorders include rheumatoid arthritis (RA), psoriasis, SLE, Alzheimer’s disease (AD), multiple sclerosis (MS), and cancers [68]. In SLE, the mechanisms responsible for the loss of immune tolerance have yet to be fully elucidated [70]; however, epigenetic factors and PTMs are becoming increasingly recognised in the pathogenesis of the disease [71]. In the context of SLE, the PTM of histone proteins, the loss of tolerance and the initiation of an autoimmune response are consistent features of the disease [72,73].

There have been considerable developments in the use of Raman spectroscopy in the biomedical field since 2013; in addition, further publications of Raman wavenumber libraries [67] have provided a useful reference for researchers when tentatively characterising the molecular assignments of Raman peaks in biofluids, such as serum. In our study, we identified eight key spectral peaks that contributed to the discrimination between SLE patients and HC, which have molecular associations with proteins, and specifically, hydrogen bond changes, amino acids, RNA and phosphorylation-associated vibrations (1002, 1113, 1155, 1286, 1346, 1408, 1452 and 1639 cm^−1^). We hypothesise that the observed peak intensity changes seen in the SLE patients may occur because of increased PTMs on a wide variety of proteins present in the serum. These Raman peaks may provide a useful biomarker for the measurement of PTMs in SLE, and thus a novel diagnostic platform for diagnosis and monitoring of disease. Furthermore, Raman spectroscopy has the potential to offer new insight into the molecular changes occurring in patients with SLE, compared to healthy controls.

Further to our proposal of PTMs resulting in Raman peak differences between SLE patients and HCs, we hypothesise that the PTM may affect dsDNA antibodies directly and could result in these antibodies having different affinities in different patients or disease phenotypes. This could have an impact both clinically and on the ability to detect these antibodies with our current armoury of laboratory tests. These modifications could also be impacted by drugs and treatment and, therefore, vary over time within the same patient. As these PTMs patterns may be reflected in the Raman signatures obtained from serum samples, this method could offer a highly detailed insight into patient status when considering clinical phenotypes, disease progression and treatment response.

An increased Raman intensity of a peak at 1070 cm^−1^ was observed in SLE patients, compared to HC. This peak has been attributed to symmetric PO_2_ stretching of DNA, representing an increase in the amount of DNA present. Two further DNA-associated peaks were identified in our study (1452 cm^−1^ and 1639 cm^−1^), which also demonstrated increased Raman intensity in the SLE group, compared to HC. The higher level of DNA in serum samples of SLE patients could result from the ineffective clearance of dying cells [74] or the release of DNA from neutrophil extracellular traps, which have failed to be removed effectively [75], both of which have been reported as pathological mechanisms in SLE. The clinical utility of measuring cell-free circulating DNA (cf-DNA) has previously been investigated in SLE patients [76]. Significantly increased levels of cf-DNA were identified in SLE patients, compared to controls, and a significant reduction in levels was noticed in response to therapy. This demonstrates a potential new disease marker and tool to monitor the response to treatment in SLE using the molecular biology technique of real time PCR to detect cf-DNA. Although this highly advanced technique is sensitive and specific, disadvantages include the high cost, complexity of sample processing and the number of components required to perform the test. Raman spectroscopy could provide a fast, label-free test with minimal sample processing required. We suggest that DNA-associated Raman peaks could be evaluated as an alternative technique to further explore the clinical utility of this diagnostic and disease monitoring test.

As the tentative molecular assignments for FTIR and Raman spectroscopy wavenumber libraries are continually evolving, it would be of great interest to collaborate with research groups specialising in the field of SLE pathogenesis and the identification of new biomarkers. Future work using a collaborative approach would aid the molecular interpretation of key wavenumbers by aligning SLE-specific research findings from groups investigating PTMs and novel markers, such as cf-DNA, with the spectral biomarkers identified in our work. We have achieved the primary aim of this study in demonstrating the proof of concept for using Raman spectroscopy in the setting of SLE. We further predict that future development and use of this technology could provide novel insights into aetiological and molecular mechanisms, underpinning not only SLE but also a wide repertoire of autoimmune rheumatological disorders.

## 5. Conclusions

Our work has echoed claims made by the literature and clinicians surrounding the variable clinical utility of anti-dsDNA antibody testing in SLE. Our audit affirmed the widely reported variability between results obtained by two routine testing platforms for dsDNA antibodies. We saw a strong association between SLE and positive dsDNA antibody results when obtained by both platforms; however, false positivity remains an issue, particularly with single platform ELIA positivity. Although there remains clinical utility with current anti-dsDNA antibody methods, given the recognised shortcomings alongside the clinical heterogeneity in SLE, there is scope for the development and standardisation of dsDNA methods.

The feasibility study of a novel use of Raman spectroscopy in SLE delivered promising results and a solid foundation for further research in this area. Multi-variant analysis revealed Raman signature differences between serum samples from healthy controls and SLE patients, highlighting detectable biological variance in SLE disease profiles. We also developed classification models capable of successfully segregating SLE patients from healthy controls, regardless of the dsDNA antibody result profile (negative, single- or dual-positive). These encouraging findings provide a platform to develop a future diagnostic test for SLE using Raman spectroscopy and multivariate analysis techniques.

## Figures and Tables

**Table 1:** Categories of SLE patients determined by autoantibody positivity combinations. Groups 1, 2 and 3 each composed of SLE patients with varying positivity for anti-nuclear and anti-dsDNA antibodies. Group 1—‘ELIA Neg CLIFT N/A’, Group 2—‘ELIA Pos CLIFT Neg’, Group 3—‘ELIA Pos CLIFT Pos’. Four healthy controls with no clinical indication of SLE or any other autoimmune disease that were negative for ANA and dsDNA by ELIA and CLIFT were included as the healthy control group (Group 0). ‘+’ = Positive, ‘-’ = Negative.

Group	ANA	ELIA dsDNA	CLIFT	Number of Samples	Total Number of Spectra
Healthy Controls—0	-	-	-	4	80
SLE—1	+	-	N/A	2	40
SLE—2	+	+	-	2	37
SLE—3	+	+	+	4	77

**Table 2:** Significant Raman peaks. The 12 most significant peaks detected by vector cluster analysis in MATLAB of healthy controls and SLE patients. Arrows indicate Raman intensity increase (↑) or decrease (↓) in SLE patients compared to HCs. Significance * (*p* < 0.001) ** (*p* < 0.0001).

Peaks (Waves/cm)	Molecular Assignment (Irootlab)	Molecular Assignment (Literature Review)	Increased/Decreased in SLE	Significance	Reference
1002	Protein Phosphorylation	(Stretching vibration) ring-breathing Phenylalanine (collagen assignment), protein	↑	**	[46,47]
1070	Protein Phosphorylation	Triglycerides (fatty acids) (1070–1090) Symmetric PO_2_—stretching of DNA (represents more DNA in cell)	↑	**	[48,49]
1113	Protein Phosphorylation	Several bands of moderate intensity, belonging to amide III and other groups (proteins) (1100–1375 cm^−1^)	↑	**	[50]
1155	Protein Phosphorylation	C-C (and C-N) stretching of proteins (also Carotenoids) Glycogen	↓	**	[51,52,53,54]
1286	Protein Phosphorylation	Amide III (arising from coupling of C-N stretching and N-H bonding; can be mixed with vibrations of side chains) (protein band) (1220–1300 cm^−1^)	↑	**	[55]
1346	Protein Phosphorylation	Several bands of moderate intensity, belonging to amide III and other groups (proteins) (1100–1375 cm^−1^)	↑	**	[50]
1408	Protein Phosphorylation	ν(C=O)O^−^ (amino acids, aspartic and glutamic acid) (1400–1430 cm^−1^)	↑	*	[56]
1452	Protein Phosphorylation	CH_2_ deformation (1437–1453 cm^−1^) CH deformation (DNA/RNA and proteins and lipids and carbohydrates) (1420–1480)	↑	**	[57]
1527	Protein Phosphorylation	C-C Carotenoid (1520–1538 cm^−1^)	↑	**	[58,59]
1596	Protein Phosphorylation	COO^−^ (1560–1600 cm^−1^)	↑	**	[60]
1639	Protein Phosphorylation	In-plane double end vibrations of bases; the spectra in this region are very sensitive to base-pairing interactions and base-stacking effects; i.e., effects of hydrogen bond formation (1620–1750 cm^−1^) Amide I (which is due mostly to the C O stretching vibrations of the peptide backbone; has been used the most for structural studies due to its high sensitivity to small changes in molecular geometry and hydrogen bonding of peptide group)	↑	**	[51,61,62]
1727	Protein Phosphorylation	C=O (1716–1741 cm^−1^)	↑	**	[63]

**Table 3:** Sensitivity, specificity, positive and negative predictive value of ELIA dsDNA and CLIFT methods. PPV—Positive predictive value; NPV—Negative predictive value.

Method	Sensitivity (%)	Specificity (%)	PPV	NPV
ELIA dsDNA	81.0	83.7	24.8	98.5
CLIFT	67.7	95.7	85.2	89.1

## Data Availability

The data (raw spectra for SLE vs. HC subjects and SLE subgroups vs. HC) reported in this paper are available at the publicly accessible data repository Figshare (DOI:10.6084/m9.figshare.21287034; accessed on 21 October 2022).
